# Lipoxin A_4_ improves cardiac remodeling and function in diabetes-associated cardiac dysfunction

**DOI:** 10.1186/s12933-024-02501-x

**Published:** 2024-11-20

**Authors:** Ting Fu, Muthukumar Mohan, Madhura Bose, Eoin P. Brennan, Helen Kiriazis, Minh Deo, Cameron J. Nowell, Catherine Godson, Mark E. Cooper, Peishen Zhao, Barbara K. Kemp-Harper, Owen L. Woodman, Rebecca H. Ritchie, Phillip Kantharidis, Cheng Xue Qin

**Affiliations:** 1 Drug Discovery Biology, Monash Institute of Pharmaceutical Sciences, Monash University, Parkville, Victoria Australia; 2 Department of Diabetes, Central Clinical School, Monash University, Melbourne, Victoria Australia; 3 Renal Electrolyte and Hypertension Division, Department of Medicine, University of Pennsylvania, Philadelphia, Pennsylvania USA; 4 Institute for Diabetes, Obesity and Metabolism, University of Pennsylvania, Philadelphia, Pennsylvania USA; 5 Diabetes Complications Research Centre, School of Medicine and Conway Institute, University College Dublin, Dublin, Ireland; 6 Baker Heart and Diabetes Institute, Melbourne, Victoria Australia; 7 Department of Pharmacology, Biomedicine Discovery Institute, Monash University, Clayton, Victoria Australia

**Keywords:** Diabetic cardiomyopathy, Cardiac inflammation, Resolution of inflammation, Cardiac fibrosis, Specialized pro-resolving lipid mediators, Lipoxin A_4_

## Abstract

**Background:**

Diabetic heart disease may eventually lead to heart failure, a leading cause of mortality in diabetic individuals. The lack of effective treatments for diabetes-induced heart failure may result from a failure to address the underlying pathological processes, including chronic, low-grade inflammation. Previous studies have reported that lipoxin A_4_ (LXA_4_), known to promote resolution of inflammation, attenuates diabetes-induced atherosclerosis, but its impact on diabetic hearts has not been sought. Thus, we aimed to determine whether LXA_4_ therapeutic treatment attenuates diabetes-induced cardiac pathology.

**Methods:**

Six-week-old male apolipoprotein E-deficient (ApoE^−/−^) mice were followed for 16 weeks after injection of streptozotocin (STZ, 55 mg/kg/day, i.p. for 5 days) to induce type-1 diabetes (T1DM). Treatment with LXA_4_ (5 μg/kg, i.p.) or vehicle (0.02% ethanol, i.p.) was administered twice weekly for the final 6 weeks. One week before endpoint, echocardiography was performed within a subset of mice from each group. At the end of the study, mice were euthanized with sodium pentobarbital (100 mg/kg i.p.) and hearts were collected for ex vivo analysis, including histological assessment, gene expression profiling by real-time PCR and protein level measurement by western blot.

**Results:**

As expected diabetic mice showed a significant elevation in plasma glycated hemoglobin (HbA_1c_) and glucose levels, along with reduced body weight. Vehicle-treated diabetic mice exhibited increased cardiac inflammation, macrophage content, and an elevated ratio of M1-like to M2-like macrophage markers. In addition, myocardial fibrosis, cardiomyocytes apoptosis and hypertrophy (at the genetic level) were evident, with echocardiography revealing early signs of left ventricular (LV) diastolic dysfunction. Treatment with LXA_4_ ameliorated diabetes-induced cardiac inflammation, pro-inflammatory macrophage polarization and cardiac remodeling (especially myocardial fibrosis and cardiomyocytes apoptosis), with ultimate improvement in cardiac function. Of note, this improvement was independent of glucose control.

**Conclusions:**

These findings demonstrated that LXA_4_ treatment attenuated the extent of cardiac inflammation in diabetic hearts, resulting in limited cardiac remodeling and improved LV diastolic function. This supports further exploration of LXA_4_-based therapy for the management of diabetic heart disease. The recent development of stable LXA_4_ mimetics holds potential as a novel strategy to treat cardiac dysfunction in diabetes, paving the way for innovative and more effective therapeutic strategies.

**Graphical Abstract:**

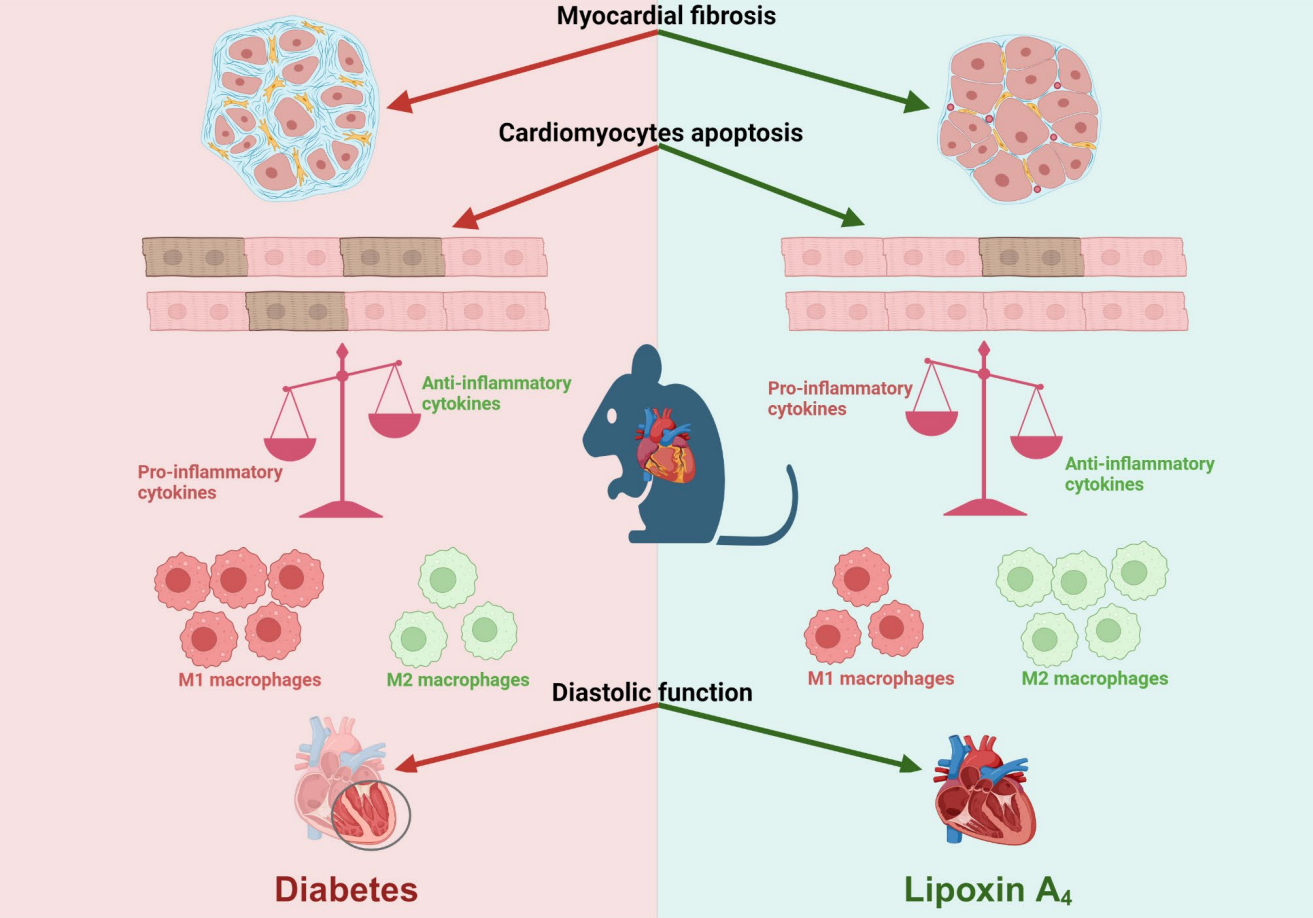

**Supplementary Information:**

The online version contains supplementary material available at 10.1186/s12933-024-02501-x.

## Introduction

Cardiovascular pathologies, such as atherosclerosis, myocardial infarction (MI), cardiomyopathy and heart failure, are leading causes of morbidity and mortality in individuals with diabetes, a major global health concern [1–3]. The diabetic heart is characterized by LV diastolic dysfunction due to cardiomyocyte hypertrophy, apoptosis, increased inflammation, and myocardial fibrosis [4, 5]. The mechanism of cardiac pathophysiology in diabetes is complex and still not fully understood. However, it includes but is not limited to, upregulated lipid oxidation, increased intramyocardial triglyceride deposition, and decreased glucose utilization, leading to higher levels of oxidative stress, mitochondrial dysfunction, and inflammation [2].

Studies of diabetic animal models and human disease have demonstrated that diabetes is associated with persistent systemic inflammation due to increased secretion of pro-inflammatory cytokines, such as the tumor necrosis factor (TNF)-α, interleukin (IL)-1β, IL-6, and IL-18 [6–8]. This supports the targeting of inflammation as a plausible therapeutic approach. As a self-protective reaction, inflammation aims to limit infection or injury and promote tissue healing. The temporal phases of successful inflammatory responses are characterized by distinct phases, including initiation and resolution. The initiation phase of an acute inflammatory response includes the release of pro-inflammatory eicosanoids [9, 10]. During the resolution phase, arachidonic acid-derived lipids undergo class switching to produce a class of specialized pro-resolving lipid mediators (SPMs), including lipoxins (LXs), resolvins, protectins, and maresins, which make an important contribution to restoring cellular homeostasis [11, 12]. Failure to resolve inflammation effectively leads to excessive tissue damage and ultimately progression to chronic low-grade inflammation, which underlies many common cardiometabolic disorders [13, 14].

Four naturally occurring LXs have been identified, i.e. LXA_4_, LXB_4_, aspirin-triggered LXA_4_ and aspirin-triggered LXB_4_ [15]. Studies have shown that LXs inhibit the activation nuclear factor κ-light-chain-enhancer of activated B cells (NF-κB) pathway in human leukocytes and reduce the release of several pro-inflammatory cytokines [16]. Brennan and colleagues also demonstrated that LXA_4_ protects against diabetic kidney disease and diabetes-associated atherosclerosis [17, 18]. The stable delivery of LXs has also been well studied. Halade and his colleagues demonstrated that the administration of aspirin-triggered LXA_4_ via liposomes promotes cardiac repair and healing, with a particular focus on modulating macrophage and fibroblast activity [19]. Based on both in vitro studies and in vivo models of MI in mice, their findings highlight the therapeutic potential of aspirin-triggered LXA_4_ in promoting post-MI healing processes through its effects on key immune and stromal cell populations. Given the growing evidence of the protective role of LXs against inflammation, promoting resolution and repair, and the inherent immunosuppression and other complications associated with anti-inflammatory, there is growing interest in developing novel therapies to promote the “resolution of inflammation” to effectively treat diabetic cardiac dysfunction.

To date, the therapeutic potential of LXA_4_, or its mimetics, on impaired cardiac phenotype and function in diabetes has not been fully explored. This study aims to test the hypothesis that treatment with the endogenous specialized pro-resolving lipid mediator, LXA_4_, attenuates cardiac inflammation, with concomitant protection against diabetes-induced cardiac remodeling and cardiac dysfunction, in mice with established T1DM.

## Methods

### Animals and experimental design

The use of mice was in accordance with protocols approved by the Alfred Medical Research and Education Precinct (AMREP) Animal Ethics Committee (E/1755/2017/B) and performed in accordance with the National Health and Medical Research Council Australia guidelines. All the animals were cared for and housed in the AMREP Precinct Animal Centre under a 12 h light/dark cycle on a standard mouse chow diet (unlimited access to water and food) (Barastoc; Ridley Agriproducts, St. Arnaud, Victoria, Australia; 20% protein, 5% fat, 6% fiber, 0.5% sodium, 0.38% ω-3 fatty acid, and 1.52% ω-6 fatty acid). The animal use flowchart was compiled by using the template from Consolidated Standards of Animal Experiment Report (CONSAERT) [20]. Age-matched, 6-week-old ApoE^−/−^ male mice (C57BL/6 background) were randomly assigned to either the diabetic or non-diabetic (ND) cohort at the start of the study. The diabetic cohort received i.p. injections of STZ (Sigma-Aldrich, St Louis, USA) for five consecutive days (55 mg/kg/day in 0.1 M citric acid vehicle). The ND cohort received the citric acid vehicle (Sigma-Aldrich, vehicle control for STZ) by i.p. injection (0.5 M). Blood glucose levels and body weight were monitored weekly after STZ injections until the end of the study. Each cohort (diabetic and ND) was randomly divided into two groups, allocated to receive either treatment vehicle (0.02% ethanol) or LXA_4_ (5 μg/kg, in 0.02% ethanol; Merck, Calbiochem, Darmstadt, Germany) twice weekly by i.p. injection [18]. Mice received LXA_4_ or treatment vehicle from week 11 to week 16 (Supplemental Figure S1 and S2).

At study endpoint, to confirm the diabetic phenotype, the following parameters were used: mice in the diabetic cohort with HbA_1c_ levels ≥ 10% and glucose levels ≥ 25 mmol/L were included in the study. For mice where the glucose levels were ≤ 26 mmol/L, if their HbA_1c_ levels were ≥ 10%, they were also included in the study (HbA_1c_ levels reflects long-term glucose control [21]).

### Echocardiography

LV function was assessed using echocardiography one week before the endpoint. Mice were anaesthetized using a ketamine/xylazine/atropine cocktail (KXA; 80/8/0.96 mg/kg i.p.). Four imaging modes [B-mode, M-mode, pulsed wave (PW) Doppler and tissue Doppler] were utilized (images obtained in that order) using a Vevo2100 imaging system and MS 550D transducer (FUJIFILM VisualSonics, Toronto, Canada). All images were analyzed by using Vevo Lab (v5.7.1) and validated by an experienced investigator (blinded) from the Preclinical Cardiology Microsurgery and Imaging Platform, Baker Heart Institute [22].

### Histology

The uppermost portion of the LV was embedded in paraffin and sections were cut (4 μm) using a Leica 2135 microtome (Leica Microsystems, Wetzlar, Hessen, Germany) and stained and scanned by the Monash University Histology Platform using an Aperio Slide scanner (Leica Microsystem, Wetzlar, Hessen, Germany). One set of sections was stained with picrosirius red to determine myocardial fibrosis (cardiac collagen content). The images (whole LV) were analyzed using a custom macro using the Fiji distribution of ImageJ (1.54f; National Institute of Health, Bethesda, Maryland, USA). In brief, the macro allowed the user to outline the regions of interest (ROI, which are the vessels in the left ventricle) for each sample. It then employed a color deconvolution and threshold function in ImageJ to automatically detect and measure the area stained by picrosirius red (Fig. 4A). The ratio of the positive stained area (highlighted in red) to the total tissue area (highlighted in green) represents the percentage of total LV collagen deposition area. The ratio of the positively stained ROI area to the total tissue area represents the percentage of collagen deposition in the perivascular region, while the ratio of the remaining positively stained area to the total tissue area represents the percentage of collagen deposition in the interstitial area. One set of sections was stained with hematoxylin and eosin (H&E) to assess cardiomyocyte size (cross-sectional area and width). The images (whole LV, 200 cardiomyocytes/slide) were analyzed using ImageJ software. One set of LV sections were stained with CardioTACS™ in situ Apoptosis Detection Kit (R&D Systems Inc, Minneapolis, Minnesota, United States) [23]. The image was taken using Leica DMi8 microscope (Leica Microsystem, Wetzlar, Hessen, Germany). TUNEL-positive nuclei and total nuclei were counted by ImageJ software.

The middle portion of the LV was frozen in Tissue-Tek Optimal Cutting Temperature compound and sectioned at 6 μm using a Microm H525 cryostat (Thermo Fisher Scientific™, Walldorf, Baden-Württemberg, Germany). Immunofluorescence staining was used to assess the number of macrophages in the myocardium. The sections were fixed with 4% paraformaldehyde (Sigma-Aldrich, St. Louis, Missouri, USA). Subsequently, 5% normal goat serum was added for blocking. Slides were incubated with either rat anti-mouse CD68^+^ (1:200, Bio-Rad, Hercules, California, USA), rat anti-mouse iNOS (1:100, Thermo Fisher Scientific™, Melbourne, Victoria, Australia), or rabbit anti-mouse CD206^+^ (1:100, Thermo Fisher Scientific™, Melbourne, Victoria, Australia), and then placed into a humidified chamber and incubated at 4 °C overnight. Next day, the slides were applied with Alexa Fluor 546 goat anti-rat IgG (1:1000, Invitrogen, Carlsbad, California, USA) or Alexa Fluor 488 goat anti-rabbit IgG (1:1000, Invitrogen, Carlsbad, California, USA). 4′,6-diamidino-2-phenylindole (DAPI; 1:1000; Invitrogen, Melbourne, Victoria, Australia) was then applied for nuclei staining. Sudan Black was added to decrease the autofluorescence, as previously described [24]. Slides were stored at 4 °C until imaged. The image (whole LV) was taken using a Nikon A1R confocal microscope (Nikon, Tokyo, Japan), with DAPI and FIC channels under 20 × magnification. 10 representative images per LV were averaged.

### Gene expression

The LV RNA extraction was performed by using a GenElute™ Mammalian Total RNA Miniprep Kit (Sigma-Aldrich, St. Louis, Missouri, USA), according to the manufacturer’s instructions. cDNA was synthesized using a High-Capacity cDNA Reverse Transcription Kit, following the manufacturer’s instructions (Thermo Fisher Scientific™, Melbourne, Victoria, Australia). Quantitative real-time polymerase chain reaction (qRT-PCR) was performed to assess genes of interest (e.g. myocardial fibrotic genes, inflammatory genes, apoptotic genes) by using SYBR® Green Real-Time PCR Mix (Applied Biosystems, Melbourne, Victoria, Australia), and primers generated from mouse-specific sequences (Supplemental Table S1). The expression of the target gene was calculated by using the comparative 2^−ΔΔCt^ method. The housekeeper gene (β-actin) was used to normalize the relative expression of target genes as described [24].

### Western blotting

As previously described, one portion of LV (~ 30 mg) was extracted using RIPA buffer [25]. A BCA protein assay kit (Sigma-Aldrich) determined the protein concentration. Diluted protein lysates (30 μg) were loaded onto 7.5% SDS-PAGE gels and transferred to a polyvinylidene difluoride membrane (Immobilon-FL, Millipore). Membranes were incubated with primary antibodies (1:1000 dilution, anti-5 lipoxygenase, Thermo Fisher; 1:1000 dilution, anti-15 lipoxygenase antibody, Abcam; 1:1000 dilution, anti-calnexin C-terminal Rabbit Polyclonal, Abcam; 1:1000 dilution, anti-collagen III antibody, Abcam; 1:200 dilution, anti-fibronectin antibody, Santa Cruz) overnight at 4 °C. The next day, the membranes were incubated with secondary antibodies (1:3000 in skim milk) for 1 h at room temperature. Images were captured using a ChemiDoc imaging system (Bio-Rad), and quantified by Image Lab software (Bio-Rad).

### Statistical analysis

The data are presented as mean ± SEM. The experimental groups (ND + vehicle, ND + LXA_4_, diabetic + vehicle, and diabetic + LXA_4_) were compared using two-way ANOVA with a Fishers LSD post hoc test and a p value of < 0.05 was considered significant. Graphs were generated and analyses were performed using GraphPad Prism*®* software version 8 (GraphPad Software, California, USA).

## Results

### Streptozotocin induces type 1 diabetes in ApoE^−/−^ mice

**Fig. 1:**
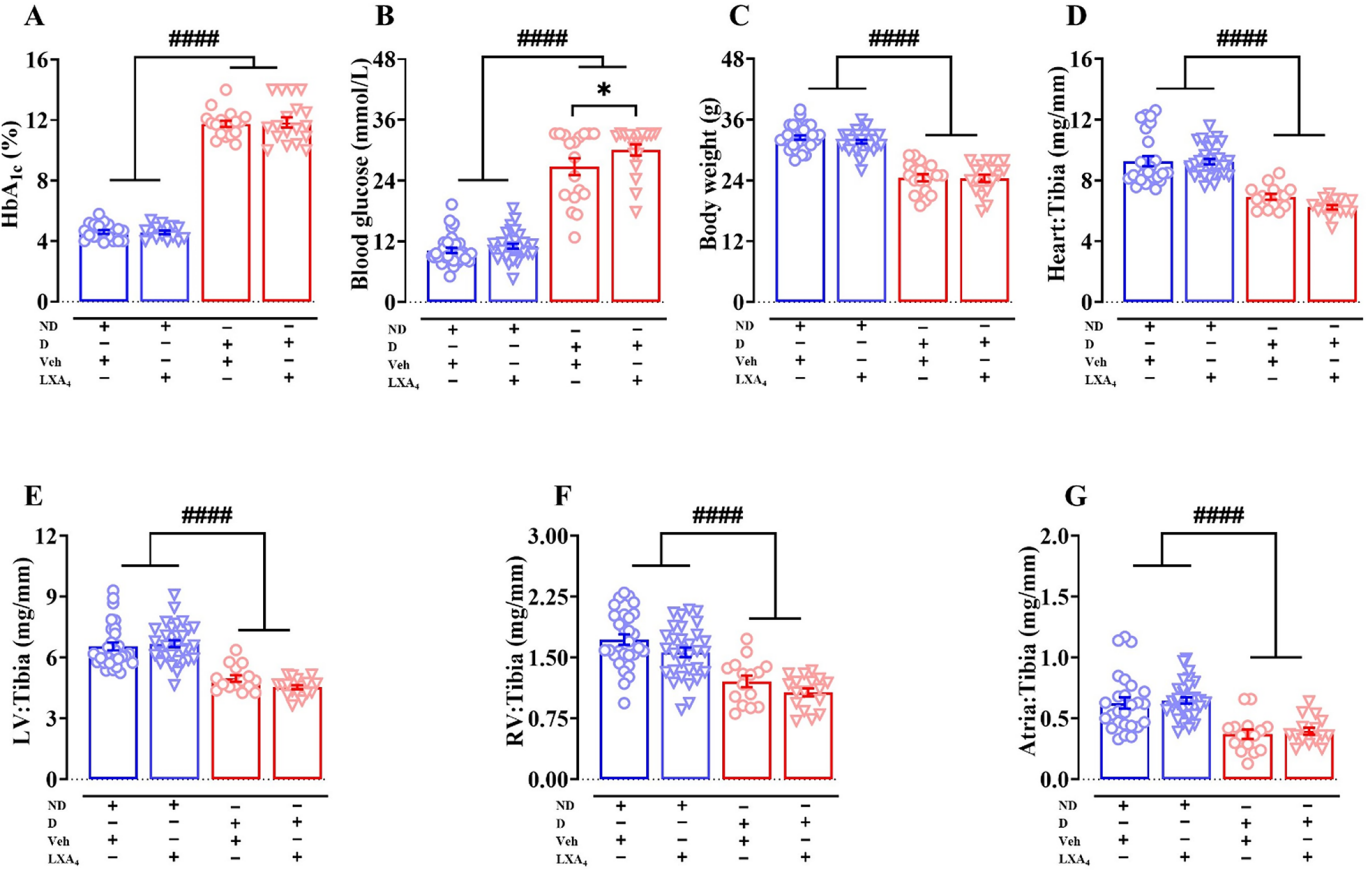
Metabolic parameters at study endpoint. **A** Endpoint HbA_1c_ levels.** B** Endpoint blood glucose levels.** C** Endpoint body weight. **D**–**G** Cardiac weight relative to tibia length. Data are presented as means ± SEM. Two-way ANOVA followed by Fisher’s LSD post hoc test was used to compare the effects of phenotype and treatment. *p < 0.05, ^####^p < 0.0001. HbA_1c_, glycated haemoglobin; LV, left ventricle; RV, right ventricle; ND, non-diabetic; D, diabetic; Veh, vehicle

This study used STZ to induce type 1 diabetes in ApoE^−/−^ mice. We followed mice for 16 weeks, in order for cardiac pathology to become established. At the study endpoint, the HbA_1c_ levels and blood glucose levels were significantly elevated in diabetic mice compared with their control (p_(phenotype)_ < 0.0001, Fig. 1A and B). The LXA_4_-treated diabetic mice exhibited slightly (but significantly) higher blood glucose levels compared to diabetic mice (30.1 ± 1.1 mmol/L vs. 26.8 ± 1.6 mmol/L, Fig. 1B). However, treatment with LXA_4_ had no impact on HbA_1c_ levels in either diabetic or control groups (Fig. 1A). Diabetic mice exhibited a lower final body weight compared to ND mice (p_(phenotype)_ < 0.0001, Fig. 1C). Administration of LXA_4_ therapy for 6 weeks had no impact on body weight (Fig. 1C). Tibia length did not alter between groups (Table 1). Relative to tibia length, the whole heart weight, LV weight, right ventricle (RV) weight and atrial weight were significantly reduced in diabetic mice (Fig. 1D–G). Taken together, these data indicate that this model (STZ treated mice studied on an ApoE^−/−^ background) is consistent with a type 1 diabetes phenotype and that LXA_4_ treatment had a minimum effect on the diabetic phenotype.

**Table 1:** Systemic characteristics at endpoint

Systemic characteristics	Non-diabetic		Diabetic	
	Vehicle	Lipoxin A_4_	Vehicle	Lipoxin A_4_
Heart (mg)	163.0 ± 5.8 (n = 28)	159.5 ± 3.2 (n = 31)	116.5 ± 2.5^##^ (n = 18)	105.9 ± 2.7^$^ (n = 16)
Left ventricle (mg)	115.2 ± 3.5 (n = 32)	115.5 ± 3.0 (n = 31)	85.2 ± 2.2^##^ (n = 18)	77.3 ± 1.9^$^ (n = 18)
Right ventricle (mg)	30.7 ± 1.3 (n = 31)	27.0 ± 1.0^#^ (n = 31)	19.5 ± 1.1^##^ (n = 18)	18.2 ± 0.8^$^ (n = 18)
Atria (mg)	11.0 ± 0.8 (n = 28)	11.2 ± 0.5 (n = 31)	6.3 ± 0.5^##^ (n = 18)	6.8 ± 0.5^$^ (n = 16)
Tibia length (mm)	17.3 ± 0.1 (n = 32)	17.3 ± 0.1 (n = 31)	16.7 ± 0.1 (n = 18)	16.9 ± 0.1 (n = 18)

^#^P < 0.05, ^##^P < 0.0001 vs non-diabetic + vehicle; ^$^P < 0.0001 vs non-diabetic + lipoxin A_4_ (2-way ANOVA, Fisher’s post hoc for multiple comparisons)

### Lipoxin A_4_ limits macrophage polarization in LV

**Fig. 2:**
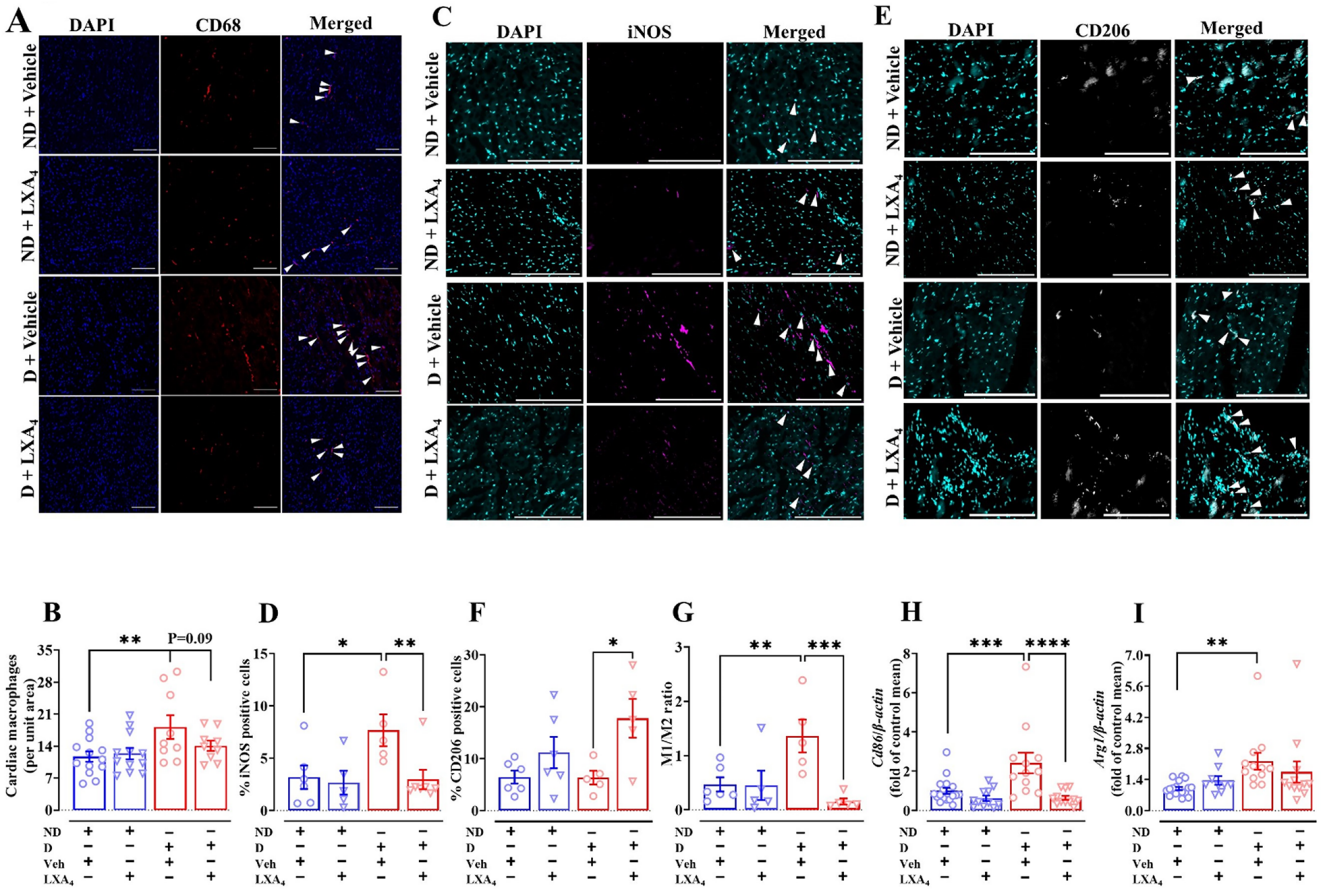
Effects of lipoxin A_4_ on macrophage polarization in LV. **A** Representative images of macrophages in the left ventricle, co-stained with nuclei (blue, DAPI) and macrophage (red, CD68). Scale bar = 200 μm. **B** Quantitative data of CD68-positive macrophages. **C** Representative images of M1-like macrophages in the left ventricle, co-stained with nuclei (cyan, DAPI) and M1-like macrophage (magenta, iNOS). Scale bar = 200 μm.** D** Quantitative data of iNOS-positive macrophages. **E** Representative images of M2-like macrophages in the left ventricle, co-stained with nuclei (cyan, DAPI) and M2-like macrophage (grey, CD206). Scale bar = 200 μm.** F** Quantitative data of CD206-positive macrophages. **G** M1/M2 macrophage ratio **H** mRNA expression of *mCd86* in mouse left ventricle. **I** mRNA expression of *mArg1* in mouse left ventricle. Data are presented as mean ± SEM, Two-way ANOVA followed by Fisher’s LSD post hoc test was used to compare the effects of phenotype and treatment. *P < 0.05, **P < 0.01, ***P < 0.001 and ****P < 0.0001. *Arg1*, arginase-1

Macrophages are the key contributors to both the initiation and the resolution of inflammation. There was a greater accumulation of CD68-positive (a marker of pan macrophages) in the LV in diabetic mice compared with the ND mice (Fig. 2A and B), which was not impacted by LXA_4_ administration (Fig. 2B). We then investigated whether LXA_4_ promotes macrophage polarization which is critical for the resolution of inflammation. We found that the mRNA expression of *Cd86* (a marker of M1-like macrophages, Fig. 2H) and arginase 1 (*mArg1*, a marker of M2-like macrophages, Fig. 2I) were upregulated in the LV of vehicle-treated diabetic mice. Notably, although no changes were detected in the mRNA expression of *mArg1* in LXA_4_-treated diabetic mice, the expression of *mCd86* significantly decreased in LXA_4_-treated diabetic mice compared with vehicle-treated diabetic mice (Fig. 2H and I). To confirm these observations, immunofluorescence staining was used to further demonstrate that the percentage of iNOS-positive cells (a marker of M1-like macrophages) was higher in vehicle-treated diabetic mice. Further, LXA_4_ significantly reduced the abundance of iNOS-positive cells in mouse LV tissues (Fig. 2C and D). The presence of CD206-positive cells (a marker of M2-like macrophages) was not different in vehicle-treated diabetic mice. However, treatment of mice LXA_4_ exhibited a higher percentage of CD206-positive cells (Fig. 2E and F). Furthermore, the M1/M2 ratio was higher in vehicle-treated diabetic mice compared to ND mice, and that this ratio was lower in LXA_4_-treated mice (Fig. 2G). These data indicate that LV macrophage accumulation is associated with diabetes and LXA_4_ may limit monocyte-macrophages polarization to M1-like (pro-inflammatory) macrophages.

### Lipoxin A_4_ attenuates LV inflammation

**Fig. 3:**
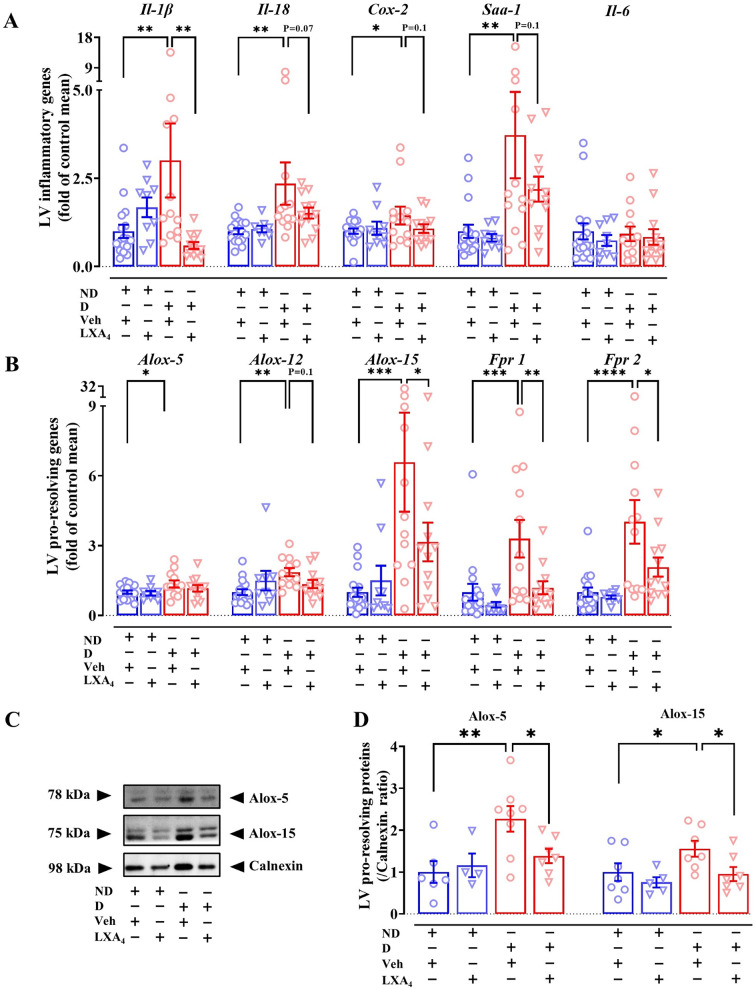
Effects of lipoxin A_4_ on cardiac inflammation in diabetic mice. **A** mRNA levels of inflammatory markers. **B** mRNA levels of pro-resolving markers. **C** Representative LV immunoblot of Alox-5, Alox-15 and calnexin. **D** LV protein expression of Alox-5 and Alox-15. Data are presented as mean ± SEM, Two-way ANOVA followed by Fisher’s LSD post hoc test was used to compare the effects of phenotype and treatment. *P < 0.05, **P < 0.01, ***P < 0.001 and ****P < 0.0001. *Il*, interleukin; *Cox*, cyclooxygenase; *Saa1*, serum amyloid A1; *Alox*, arachidonate lipoxygenase; *Fpr*, formylpeptide receptor

Cytokines play important roles in inflammation, such as in the regulation of cell proliferation, migration and adhesion. Relevant to inflammatory markers, we observed an upregulation of *mIl-1β*, *mIl-18,* cyclooxygenase-2 (*mCox-2*) and serum amyloid A1 (*mSaa1*) in vehicle-treated diabetic mice compared with ND mice (Fig. 3A). LXA_4_ treatment of diabetic mice reduced the expression of *mIl-1β*, and tended to downregulate *mIl-18*, *mCox-2* and *mSaa1*. There was, however, no difference in *mIl-6* expression between the groups (Fig. 3A). Lipoxin-synthesizing arachidonate 5/12/15-lipoxygenase (Alox-5/12/15) is the enzyme responsible for the biosynthesis of pro- and anti-inflammatory lipid mediators. The expression of *mAlox-5, mAlox-12* and *mAlox-15* in the vehicle-treated diabetic group was significantly upregulated compared to the ND group (Fig. 3B). Treatment with LXA_4_ showed a tendency toward decreased *mAlox-12* expression and a significant downregulation of *mAlox-15* expression compared to the diabetic group (Fig. 3B). We also confirmed Alox-5 and Alox-15 protein levels were elevated in vehicle-treated diabetic group compared to the ND group (Figs. 3C and D). Furthermore, treatment with LXA_4_ reduced Alox-5 and Alox-15 protein levels (Fig. 3C and D). The expression of formylpeptide receptors (FPR1 and FPR2), which are involved in both pro and anti-inflammatory responses, involved in both pro- and anti-inflammatory responses) expression were also assessed using qRT-PCR analysis. Vehicle-treated diabetic mice exhibited an upregulated expression of *mFpr1* and *mFpr2* (Fig. 3B), and when the mice were treated with LXA_4_, the expression of both receptor transcripts was significantly downregulated (Fig. 3B).

### Lipoxin A_4_ alleviates LV fibrosis in diabetic mice

**Fig. 4:**
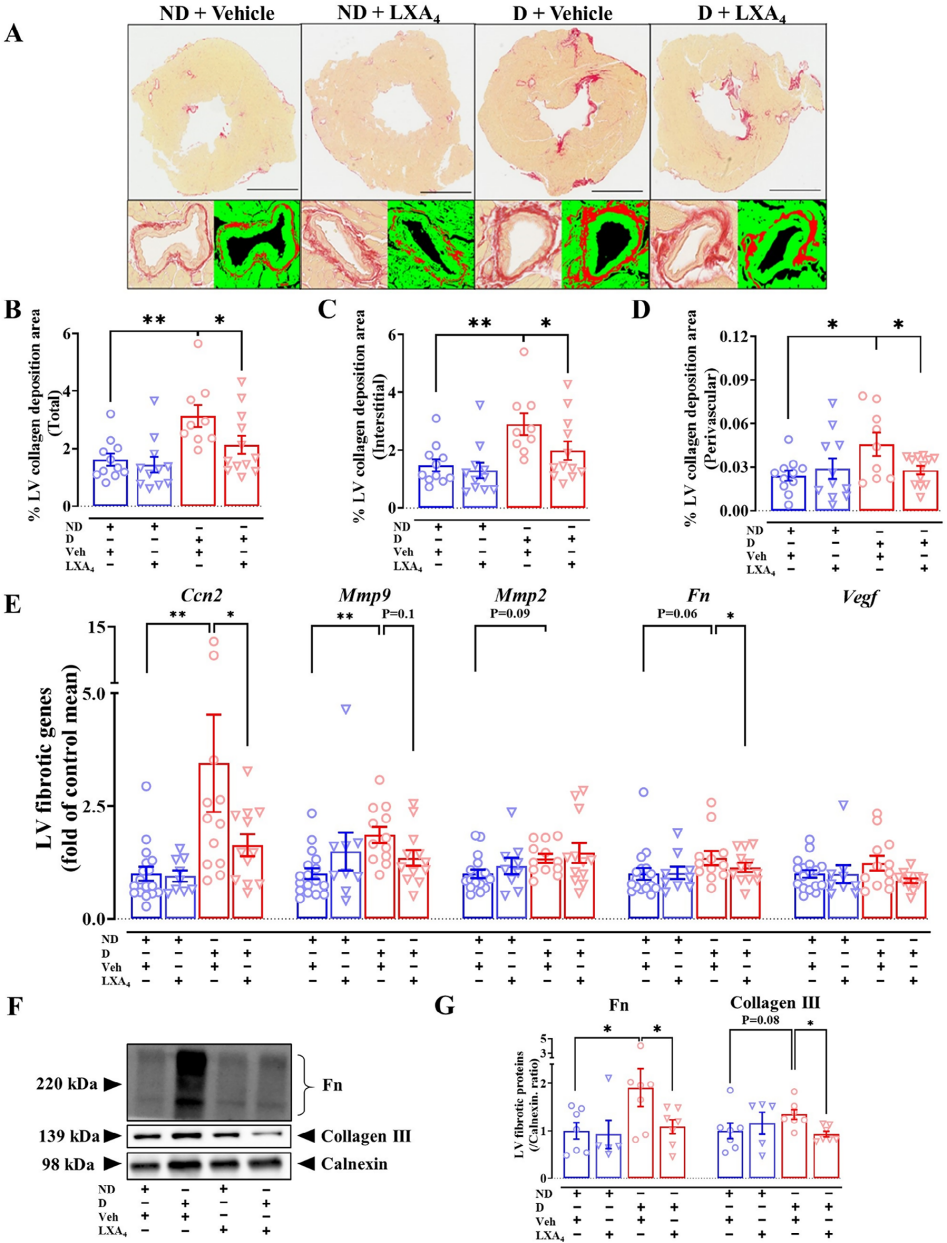
Effects of lipoxin A_4_ on cardiac fibrosis in diabetic mice. **A** Representative image of the left ventricle with picrosirius red staining. Scale bar = 1 mm. **B**–**D** Quantification of the entire left ventricle, interstitial and perivascular collagen deposition in the indicated groups. **E** mRNA levels of fibrotic markers. **F** Representative LV immunoblot of fibronectin, collagen III and calnexin. **G** LV protein expression of fibronectin and collagen III. Data are presented as mean ± SEM. Two-way ANOVA followed by Fisher’s LSD post hoc test was used to compare the effects of phenotype and treatment. *P < 0.05, **P < 0.01. *Ccn2*, connective tissue growth factor; *Mmp9*, matrix metallopeptidase 9; *Mmp2*, metallopeptidase 2; *Fn*, fibronectin; *Vegf*, vascular endothelial growth factor

Cardiac fibrosis is a key structural feature of the diabetic heart that is evident both in individuals with diabetes (with and without HF) and in pre-clinical animal models of diabetes [2, 26, 27]. There was a significant increase in total LV collagen deposition in vehicle-treated diabetic mice, compared with ND mice (Fig. 4A and B). Importantly, LV interstitial and perivascular collagen levels were significantly increased in vehicle-treated diabetic mice (Fig. 4C and D). Treatment of diabetic mice with LXA_4_ significantly reduced the collagen content in all assessed regions of the LV (Fig. 4B–D). Consistent with this histological data, we found that the mRNA level of the pro-fibrotic genes, such as connective tissue growth factor (*mCcn2*) and matrix metallopeptidase 9 (*mMmp9*) in the LV, was upregulated in diabetic mice compared with ND mice (Fig. 4E). Other fibrotic genes such as *mMmp2* and fibronectin (*mFn*) showed a tendency towards upregulation in diabetic mice (Fig. 4E), while the expression of vascular endothelial growth factor (*mVegf*) was not affected. *mCcn2* and *mFn* were significantly downregulated in LXA_4_-treated diabetic mice (Fig. 4E), and there was a tendency towards downregulating of *mMmp9* in the LXA_4_-treated diabetic group. Furthermore, LV protein expression of fibronectin was significantly higher in diabetic mice, while collagen III exhibited a trend toward increased expression in diabetic mice compared to ND mice (Fig. 4F and G). These findings suggest enhanced extracellular matrix remodeling in the diabetic group, consistent with the pathological alterations observed in diabetic cardiomyopathy, with LXA_4_ attenuating collagen deposition in the LV of diabetic mice.

### Lipoxin A_4_ attenuates LV cardiomyocyte hypertrophy and apoptosis

**Fig. 5:**
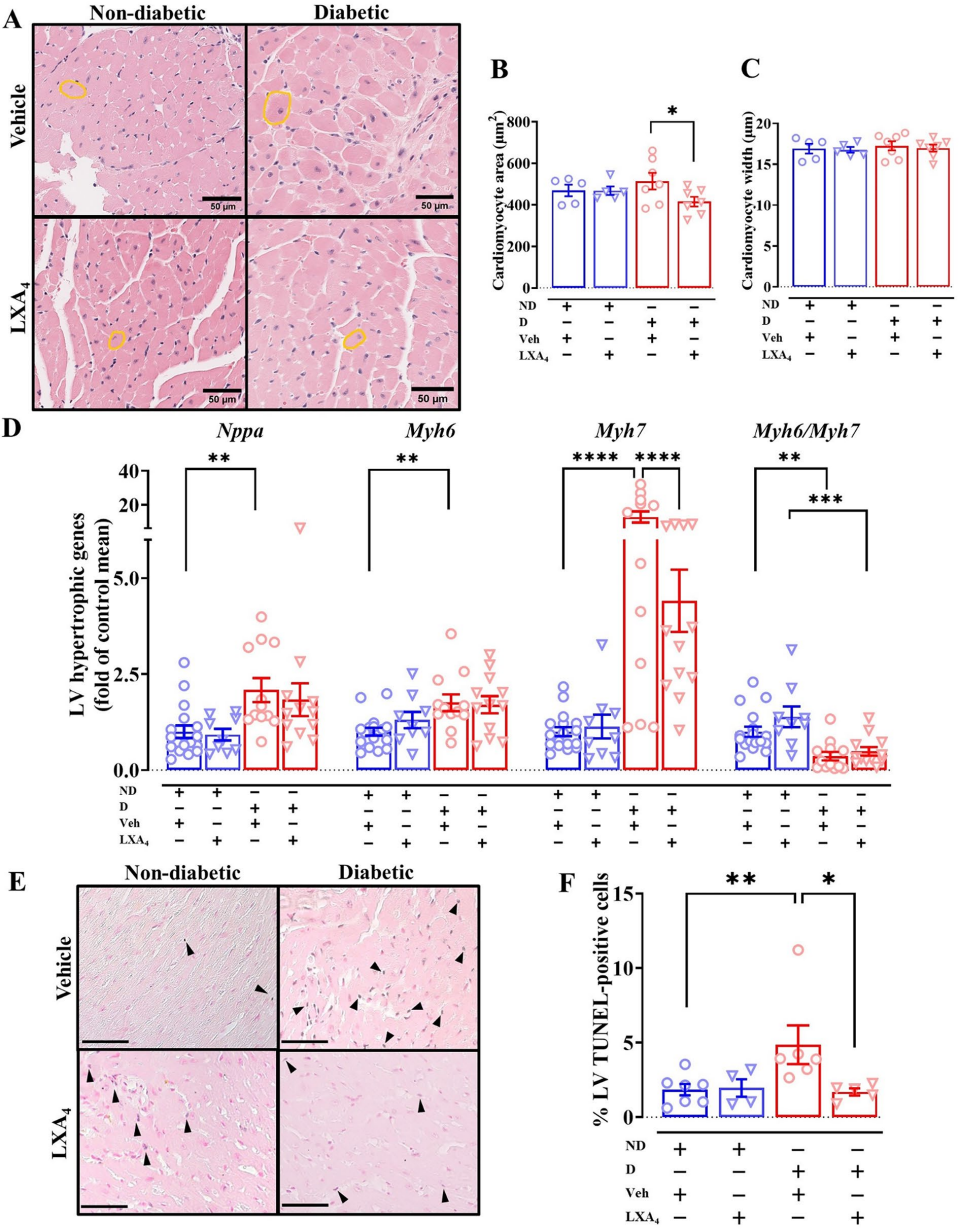
Effects of lipoxin A_4_ on cardiac hypertrophy and cardiomyocyte apoptosis in diabetic mice. **A** mRNA levels of hypertrophic markers. **B** Representative images of the left ventricle with H&E staining. Scale bar = 50 µm. **C** and **D** Quantification of cardiomyocyte area and width in the indicated groups. **E** Representative images of the left ventricle with TUNEL staining, scale bar = 100 µm.** F** Quantification of the percentage of apoptotic cardiomyocytes (blue and pink co-stained nuclei) to total cells (pink-stained nuclei). Data are presented as mean ± SEM. Two-way ANOVA followed by Fisher’s LSD post hoc test was used to compare the effects of phenotype and treatment. *P < 0.05, **P < 0.01, ***P < 0.001 and ****P < 0.0001. *Nppa*, natriuretic peptide A; *Myh7*, β-myosin heavy chain; *Myh6*, α-myosin heavy chain

Cardiomyocyte hypertrophy is one of the pathological changes commonly observed in diabetic cardiomyopathy [2]. Cardiac-specific biomarkers for cardiac hypertrophy (e.g. β-myosin heavy chain and atrial natriuretic peptide) are valuable for assessing cardiac remodeling and heart failure. Moreover, there are reports that, in heart failure, the α-myosin heavy chain and β-myosin heavy chain ratio (*mMyh6*/*mMyh7*) is decreased in animal models [28]. In the present study we show that diabetic mice had a significantly elevated expression of atrial natriuretic peptide A (*mNppa*), *mMyh6* and *mMyh7*, as well as a decreased *mMyh6/mMyh7* ratio (Fig. 5A). No change in mRNA expression of *mNppa* was observed in mice treated with LXA_4_, but the expression of *mMyh7* was significantly downregulated in LXA_4_-treated diabetic mice compared to vehicle-treated diabetic mice (Fig. 5A). We performed H&E staining to measure the cross-sectional area and width of cardiomyocytes as a measure of hypertrophy (Fig. 5B). The cross-sectional area and width were not different in diabetic mice compared to ND mice (Fig. 5C and D), but treatment with LXA_4_ appeared to reduce the area (but not the 2D width) of cardiomyocytes, compared to vehicle-treated mice (Fig. 5C). These findings suggest that our model showed a mild cardiac hypertrophy phenotype at the gene expression level, and LXA_4_ may attenuate the development of cardiac hypertrophy. Moreover, a higher number of TUNEL-positive cells was evident in diabetic mice, compared to ND mice (Fig. 5E and F). Treatment with LXA_4_ showed fewer TUNEL-positive cells in the LV (Fig. 5F). Taken together, the higher number of apoptotic cells in the LV in our model is consistent with the previous study using STZ-induced T1DM mice [29]. Additionally, LXA_4_ may protect against cardiomyocyte apoptosis.

### Lipoxin A_4_ improves LV diastolic function

**Fig. 6:**
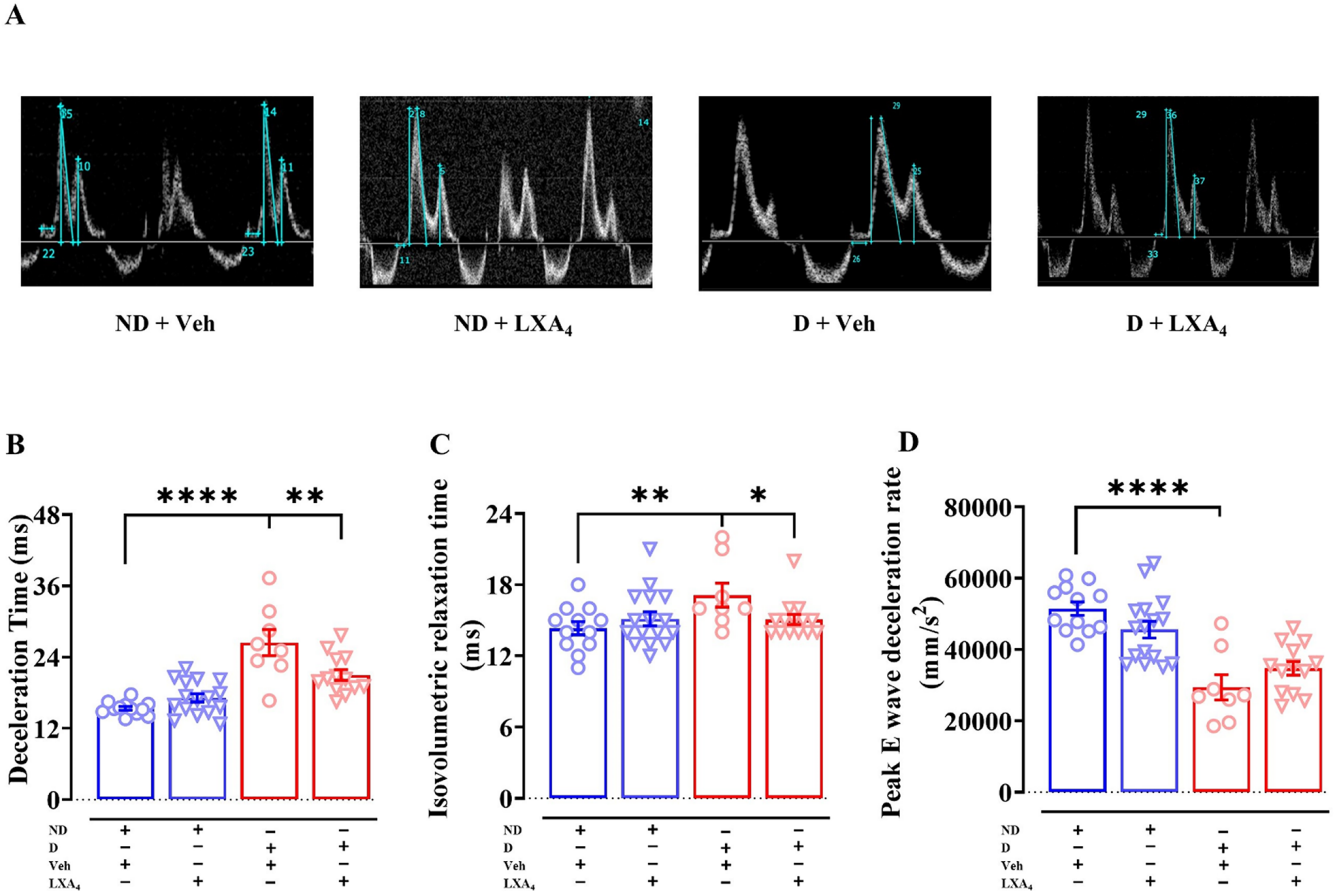
Effects of Lipoxin A_4_ on diastolic function in diabetic mice. **A** Representative Doppler image from each group **B** Deceleration time. **C** Isovolumetric relaxation time. **D** Rate of E-wave deceleration. Data are presented as mean ± SEM, n = 8–16. Two-way ANOVA followed by Fisher’s LSD post hoc test was used to compare the effects of phenotype and treatment; *P < 0.05, **P < 0.01, ***P < 0.001, ****P < 0.0001

PW Doppler echocardiography was performed to assess LV diastolic function (Fig. 6A). Vehicle-treated diabetic mice exhibited a prolonged deceleration time (DT, approximately 11 ms) compared to vehicle-treated ND mice (Fig. 6B***, ***Table 2). Treatment with LXA_4_ shortened the DT compared to vehicle-treated diabetic mice, but did not restore it to the level observed in ND (Fig. 6B). Furthermore, isovolumetric relaxation time (IVRT), another measure of LV diastolic function, was significantly prolonged in vehicle-treated diabetic mice compared to ND mice (Fig. 6C). The IVRT was restored to ND levels by LXA_4_ in diabetic mice (Fig. 6C). The rate of E-wave deceleration of vehicle-treated diabetic mice was slower than vehicle-treated ND mice, but this was not impacted by LXA_4_ treatment in either ND or diabetic mice (Fig. 6D). Diabetic mice showed a lower LV volume, area and cardiac output, compared to ND mice (Supplemental Tables S2). There were no significant differences between vehicle treated ND mice and vehicle treated diabetic mice in LV posterior wall diastolic thickness (PWd), LV anterior wall diastolic thickness (AWd), LV end-systolic dimension (LVESD), LV end-diastolic dimension (LVEDD) and fractional shortening assessed by M-mode echocardiography. However, compared to LXA_4_ treated ND mice, there is a thinner PWd, LVESD, LVEDD and a higher fractional shortening in LXA_4_ treated diabetic mice (Supplemental Tables S3). Some parameters show greater variability perhaps due to the lower sample size in vehicle-treated diabetic mice. Collectively, these data suggest that STZ-induced diabetic mice on an ApoE^−/−^ background exhibit mild diastolic dysfunction. Notably, treatment with LXA_4_ appears to partially ameliorate this dysfunction.

**Table 2:** Assessment of LV diastolic function via PW Doppler and tissue Doppler echocardiography

	Non-diabetic mice		Diabetic mice	
	Vehicle	LXA_4_	Vehicle	LXA_4_
	PW Doppler and tissue Doppler echocardiography			
N	12	16	8	13
Heart rate (bpm)	434 ± 9	421 ± 8	384 ± 12^#^	427 ± 8**
Peak E wave velocity (mm/s)	788 ± 24	760 ± 18	731 ± 38	709 ± 18
Peak A wave velocity (mm/s)	505 ± 32	467 ± 15	405 ± 21^	427 ± 10
Peak e’ velocity (mm/s)	23.9 ± 1.1	25.0 ± 0.7	23.4 ± 2.3	20.7 ± 1.3^$^
Peak a’ velocity (mm/s)	22.6 ± 1.1	21.4 ± 0.7	20.5 ± 0.9	21.5 ± 0.6
E/A ratio	1.62 ± 0.08	1.64 ± 0.05	1.83 ± 0.14	1.67 ± 0.06
e’/a’ ratio	1.09 ± 0.09	1.19 ± 0.05	1.15 ± 0.14	0.95 ± 0.06^$^
E/e’ ratio	33.7 ± 2.0	30.8 ± 1.1	32.4 ± 1.8	35.7 ± 2.1^$^
Deceleration time (ms)	15.4 ± 0.3	17.1 ± 0.7	26.4 ± 2.2^##^	21.0 ± 0.9**^, $$^
Isovolumetric relaxation time (ms)	14.3 ± 0.6	15.1 ± 0.6	17.1 ± 1.0^#^	15.1 ± 0.4*

^#^P < 0.01, ^##^P < 0.0001 vs non-diabetic + vehicle; *P < 0.05, **P < 0.01 vs diabetic + vehicle; ^$^P < 0.05, ^$$^P < 0.001 vs non-diabetic + lipoxin A_4_ (2-way ANOVA, Fisher’s post hoc for multiple comparisons)

## Discussion

This study demonstrated that ApoE^−/−^ diabetic mice exhibited cardiac inflammation, myocardial fibrosis, cardiomyocyte apoptosis, increased gene expression associated with cardiomyocyte hypertrophy, and early signs of LV diastolic dysfunction. Notably, the inflammatory response was associated with an increased number of macrophages in the heart, particularly with an increase in the M1-like macrophage population, which was also consistent with the elevated LV expression of pro-inflammatory mediators. Moreover, the expression of *mFpr1* and *mFpr2* was also increased in the diabetic heart. Our study demonstrated for the first time that chronic treatment with LXA_4_ protected against cardiac fibrosis, cardiomyocyte apoptosis and cardiomyocyte hypertrophy. Furthermore, a significantly reduced expression of some pro-inflammatory cytokines and *mFprs* was observed, and these changes were associated with improved in LV function.

Our current data indicate that LXA_4_ does not significantly impact glucose control, suggesting that it may not influence the synthesis of ketone bodies. However, Börgeson et al. have demonstrated that lipoxin A_4_ and its analogue, Benzo-LXA_4_, can limit liver expansion, reduce serum alanine aminotransferase levels, and decrease hepatic triglyceride levels in models of obesity-induced liver disease [30]. However, the effects of LXA_4_ on ketone body metabolism remain largely unknown, particularly in the context of diabetic ketoacidosis. Further investigation is needed to elucidate the potential interactions between LXA_4_ and ketone body dynamics in this pathological state.

Our study employed robust T1DM models, pharmacologically induced by STZ [2]. This model not only replicates diabetic-induced fibrosis, apoptosis, hypertrophy and cardiac inflammation, but also exhibits the functional phenotype, especially diastolic dysfunction, mirroring the pathological features observed in the diabetic human heart [31]. Given that chronic low-grade inflammation plays an important role in the development of diabetic cardiomyopathy [32, 33], we utilized ApoE^−/−^ mice on a C57BL/6 background in this study. Due to their deficiency in ApoE, even on a regular diet, these mice demonstrate elevated cholesterol levels, macrophage accumulation, and increased release of cytokines and chemokines, resulting in heightened systemic inflammation [34]. These characteristics provide a valuable platform for studying chronic low-grade inflammatory diseases, including cardiovascular and metabolic disorders [35].

A previous study in our laboratory has demonstrated that diabetes increases cardiac macrophage content, which was evident in diabetic mice 8 weeks post STZ-induction, and this persisted beyond 16 weeks of diabetes, at least on an FVB background [31]. In the present study, an increased cardiac macrophage number, particularly M1-like macrophages, was confirmed by histological analysis in the diabetic heart. The total number of pan macrophages in the heart was not affected by LXA_4_. However, our observations suggest that LXA_4_ may limit the polarization of recruited monocyte macrophages towards an M1-like phenotype, which may have important implications for the pathophysiology of the diabetic heart. Following heart injury or other inflammatory cardiac insults, circulating monocytes can infiltrate the heart and polarize towards the M1-like (pro-inflammatory) macrophage phenotype, as previously reported in both animal models and people with diabetes [36, 37]. Our results demonstrated that M1-like macrophages were upregulated in diabetic mice, while M2-like macrophages were not. Treatment with LXA_4_ reduced the number of M1-like macrophages, and increased the number of M2-like macrophages, consistent with previous reports [30, 38].

FPR1 and FPR2 play critical roles in both the induction and resolution of inflammation[39–41]. FPR2 is the primary target for LXA_4_ and has multiple roles in cardiometabolic diseases including in aging and obesity [42]. The current study showed a similar upregulation of expression of *mFpr1* and *mFpr2* in the vehicle-treated diabetic mice, as reported in other chronic inflammatory diseases [43–45]. The induction and resolution of inflammatory responses are not only receptor-dependent, but also highly ligand-dependent, as highlighted in recent reviews [41, 46]. In chronic inflammatory conditions, such as T1DM and T2DM, several animal models have demonstrated reduced plasma levels of LXA_4_ and annexin A1 [46]. This is consistent with human data, where individuals with diabetes exhibit lower circulating levels of LXA_4_ and annexin A1 when compared to healthy controls [47]. A cohort study further revealed that diminished LXA_4_ levels are positively associated with the onset of T2DM [48]. These findings suggest that the resolution phase of inflammation may be overwhelmed by persistent inflammatory stimuli in diabetic conditions. The current study showed an increased gene expression of *mSaa1* (pro-inflammatory endogenous *mFpr2* ligand) in the vehicle-treated diabetic mice, and a similar upregulation of *mFpr1* and *mFpr2* was observed. Taken together, the diabetes-induced increase in the expression of *mFpr1* and *mFpr2* suggests an enhanced opportunity for pro-inflammatory ligands to engage these receptors, potentially exacerbating the inflammatory response. Interestingly, treatment with LXA_4_ significantly attenuated the diabetes-induced increase in *mFpr1* and *mFpr2* expression, as well as downregulating *mSaa1*, consistent with the observations of Aubeux and colleagues who reported that the gene expression of FPR2 was downregulated in M2-like macrophages [38]. Our data suggest that reduced *mFpr2* expression may be related to the phenotype switching of macrophages in the LV. However, we could not obtain unequivocal results with a commercially available antibody.

ALOX-5, 12 and 15 are critical inflammatory enzymes and it has been demonstrated that the location of ALOX-5 within leukocytes is vital for its function. For the biosynthesis of leukotriene B_4_, a pro-inflammatory lipid mediator, ALOX-5 is located in the nuclear envelope. However, for the biosynthesis of LXA_4_, a pro-resolving lipid mediator, ALOX-5 is located in the cytoplasm [49, 50]. Moreover, it has been shown that ALOX-5 was more highly expressed in M1-like macrophages than in M2-like macrophages [51]. Our study demonstrated a higher expression of Alox-5 in diabetic mice, while treatment with LXA_4_ showed a lower protein level of Alox-5. Taken together, the lower expression of Alox-5 may be related to a lower number of M2-like macrophage in diabetic mice treated with LXA_4_. LXA_4_ may also affect the intercellular localization of ALOX-5 [49, 50], which could impact the biosynthesis of lipid mediators, but this requires further investigation. In the current study, *mAlox12 and 15,* exhibited upregulation in the vehicle-treated diabetic cohort, consistent with previous studies in other diabetes-associated comorbidities [52, 53]. Overexpression of 12/15-LOX in a transgenic mice model revealed an increased cardiac macrophage content, a high level of cardiac inflammation, cardiac fibrosis and LV systolic dysfunction [54]. A novel observation in this study was that treatment with LXA_4_ blunted the LV gene expression of *mAlox12* and *15* in diabetic mice and Alox-15 protein levels.

Chronic inflammation is a primary basis for cardiac pathobiology, as a result of poorly-balanced diets in addition to deficiencies in both exercise and sleep habits [55, 56]. Cardiac fibrosis, including increased collagen deposition can result in LV diastolic dysfunction by stiffening the myocardium [2]. In this study, we observed a apparent increase in myocardial interstitial and perivascular collagen deposition, consistent with previous observations in both T1DM and T2DM in mice [53, 57, 58] and biopsies of human hearts [59]. In the current study, treatment with LXA_4_ limited the cardiac fibrosis in both myocardial interstitial and perivascular areas. Cardiomyocyte apoptosis is well recognized in both animals and individuals with diabetes [2, 4, 60]. In this study, we found a significantly higher number of apoptotic cardiomyocytes in diabetic mice, and a lower number of apoptotic cardiomyocytes following the administration of LXA_4_.

LV diastolic dysfunction is commonly noted as one of the earliest functional abnormalities, observed in the progression of diabetic heart disease [2]. In the present study, there were some early signs of impaired LV diastolic function (prolonged times for deceleration and isovolumetric relaxation) in our vehicle-treated diabetic mice, although not all of the parameters of diastolic function were impaired. The increased time for deceleration in the diabetic animals was normalized in the LXA_4_ treated diabetic animals. The differences observed between our study and previous reports may be related to different mouse strains. Over 6 weeks in diabetic ApoE^−/−^ mice, the attenuation of inflammatory responses by LXA_4_ may not sufficiently translate to a measurable improvement in cardiac function. A longer treatment regimen should be explored to fully assess the therapeutic potential of LXA_4_, provided that the stability and viability of this animal model can be optimized. Extending the duration of LXA_4_ administration may reveal more pronounced effects on cardiac function and better simulate the resolution of inflammatory responses to limit chronic inflammation in diabetic cardiomyopathy.

## Limitations and future direction

The model used in this study provides valuable insight into how macrophages play a crucial role in the progression of diabetic cardiomyopathy. It further highlights how chronic inflammation may lead to cardiac remodeling and early signs of cardiac dysfunction, which could represent a mild or early stage of diabetic heart disease. However, this model does not fully represent the pathophysiology of patients suffering from diabetic cardiomyopathy, so other models may be required to confirm the protective effects of LXA_4_. We exclusively used male mice as a proof-of-concept study, and future research should incorporate female mice, especially given that females are more susceptible to diabetic cardiovascular complications [61], and that there are observed sex-specific differences in the biosynthesis of SPMs [62]. Including both sexes will provide a more comprehensive understanding of the pathophysiological mechanisms and therapeutic responses. Previous studies have shown that with every percentage increase in HbA_1c_ levels, the risk of heart failure increases by 30% in T1DM and 8% in T2DM [63, 64]. Compared to healthy subjects, individuals with T1DM develop heart disease 10 to 15 years earlier [65]. As mentioned earlier, both T1DM and T2DM share certain pathological features of diabetic cardiomyopathy. In this study, we used a T1DM model to test the therapeutic potential of LXA_4_. However, using STZ-induced models may introduce toxic effects that influence cardiac phenotype, and may not mimic some specific pathological features of T2DM patients. Studies have shown that there are differences in cardiac remodeling and function between T1DM and T2DM-induced heart disease. For example, in T1DM (STZ-induced diabetes) animal models, mitochondrial dysfunction, inflammation, fibrosis and apoptotic pathways were more affected than control mice. In contrast, in T2DM (induced by STZ and a high-fat diet) animals, mice exhibited impaired glucose metabolism, nitric oxide signaling, and CXR4 signaling pathways compared to the control (normal chow diet) [21]. These differences may contribute to the different phenotypes of heart failure in the two models (heart failure with reduced ejection fraction for T1DM and heart failure with preserved ejection fraction for T2DM) [29]. Therefore, it is important to validate the therapeutic effects of LXA_4_ in alternative T1DM models (e.g., BioBreeding diabetes-prone mice), as well as in T2DM (e.g., *db/db* mice) [66, 67]. Moreover, the dynamic nature of the inflammatory response poses a challenge in evaluating the therapeutic efficacy of anti-inflammatory agents at a single time point in chronic inflammatory disease models. Future studies may include assessment of the inflammatory response at earlier stages (e.g., 4 weeks after the onset of diabetes) to capture the temporal dynamics of inflammation and better understand the therapeutic window for LXA_4_ [31]. Use of genetically modified animals (silenced receptor, etc.) that can demonstrate the role of FPR2 in the progression of diabetic cardiomyopathy, and further confirm targeting resolution of inflammation is a viable approach for treating diabetic cardiomyopathy. We attempted to quantify the cardiac concentration of LXA_4_; however, our measurements fell below the detectable limit. This limitation is likely attributable to the sensitivity of the analytical equipment used and the stability of LXA_4_ under the experimental conditions. Given the short half-life of lipoxin, an analysis of the plasma and cardiac concentration of LXA_4_ is needed [68]. Also, future translational studies will benefit from developing more stable lipoxin analogues. These analogues will enable a more accurate assessment of therapeutic efficacy, facilitating the translation of preclinical findings to clinical applications [69, 70].

## Conclusions

In conclusion, our study provides compelling evidence that diabetic ApoE^−/−^ mice exhibit hallmark features of diabetic cardiomyopathy (such as adverse remodeling and diastolic dysfunction). Notably, treatment with LXA_4_ significantly mitigated diabetes-induced cardiac structural remodeling, as evidenced by reduced cardiac fibrosis, decreased the number of apoptotic cardiomyocytes and downregulation of hypertrophy-related genes. LXA_4_ also ameliorated early-stage left ventricular diastolic dysfunction. Mechanistically, LXA_4_ selectively reduced the prevalence of M1-like macrophages without altering the overall macrophage population in the diabetic heart. Furthermore, LXA_4_ downregulated the expression of both FPR subtypes, correlating with decreased levels of pro-inflammatory mediators. These findings suggest that LXA_4_ could serve as a valuable adjunct to current standard-of-care therapies, potentially enhancing clinical outcomes for patients with diabetic heart disease.

## Electronic supplementary material

Below is the link to the electronic supplementary material.

Supplementary Material 1

## Data Availability

No datasets were generated or analysed during the current study.

## Abbreviations

Alox 5/12/15: Arachidonate 5/12/15-lipoxygenase
ApoE^−^^/^^−^: Apolipoprotein E-deficient
Arg1: Arginase-1
AWd: Anterior wall diastolic thickness
Ccn2: Connective tissue growth factor
Cox: Cyclooxygenase
DT: Deceleration time
EF: Ejection fraction
Fn: Fibronectin
FPR: Formylpeptide receptor
FS: Fractional shortening
HbA_1c_: Glycated hemoglobin
H&E: Hematoxylin and eosin
IL: Interleukin
i.p.: Intraperitoneal injection
IVRT: Isovolumetric relaxation time
LV: Left ventricle
LVEDD: LV end-diastolic dimension
LVESD: LV end-systolic dimension
LXs: Lipoxins
LXA_4_: Lipoxin A_4_
LXB_4_: Lipoxin B_4_
MI: Myocardial infarction
Mmps: Matrix metalloproteinases
Myh: Myosin heavy chain
ND: Non-diabetic
NF-κB: Nuclear factor κ–light-chain-enhancer of activated B cells
Nppa: Natriuretic peptide A
KXA: Ketamine/xylazine/atropine
PW Doppler: Pulsed wave Doppler
PWd: Posterior wall diastolic thickness
qRT-PCR: Quantitative real-time polymerase chain reaction
RV: Right ventricle
Saa: Serum amyloid A
SPMs: Specialized pro-resolving lipid mediators
STZ: Streptozotocin
T1DM: Type 1 diabetes mellitus
TNF-α: Tumor necrosis factor-α
Vegf: Vascular endothelial growth factor
