# Supplementary material for: Lipoxin A4 improves cardiac remodeling and function in diabetes-associated cardiac dysfunction
Source: Cardiovasc Diabetol. 2024 Nov 20;23:413. doi: 10.1186/s12933-024-02501-x (PMC11577589; doi:10.1186/s12933-024-02501-x)
Supplement: Supplementary file 1 — Supplementary Material 1 [file 12933_2024_2501_MOESM1_ESM.docx]

SUPPLEMENTAL MATERIALS

**Lipoxin A_4_ improves cardiac remodeling and function in diabetes-associated cardiac dysfunction.**

**Authors**

Ting Fu, Muthukumar Mohan, Madhura Bose, Eoin P. Brennan, Helen Kiriazis, Minh Deo, Cameron J. Nowell, Catherine Godson, Mark E. Cooper, Peishen Zhao, Barbara K. Kemp-Harper, Owen L Woodman, Rebecca H Ritchie, Phillip Kantharidis and Cheng Xue Qin.

**1. Supplemental Figures**


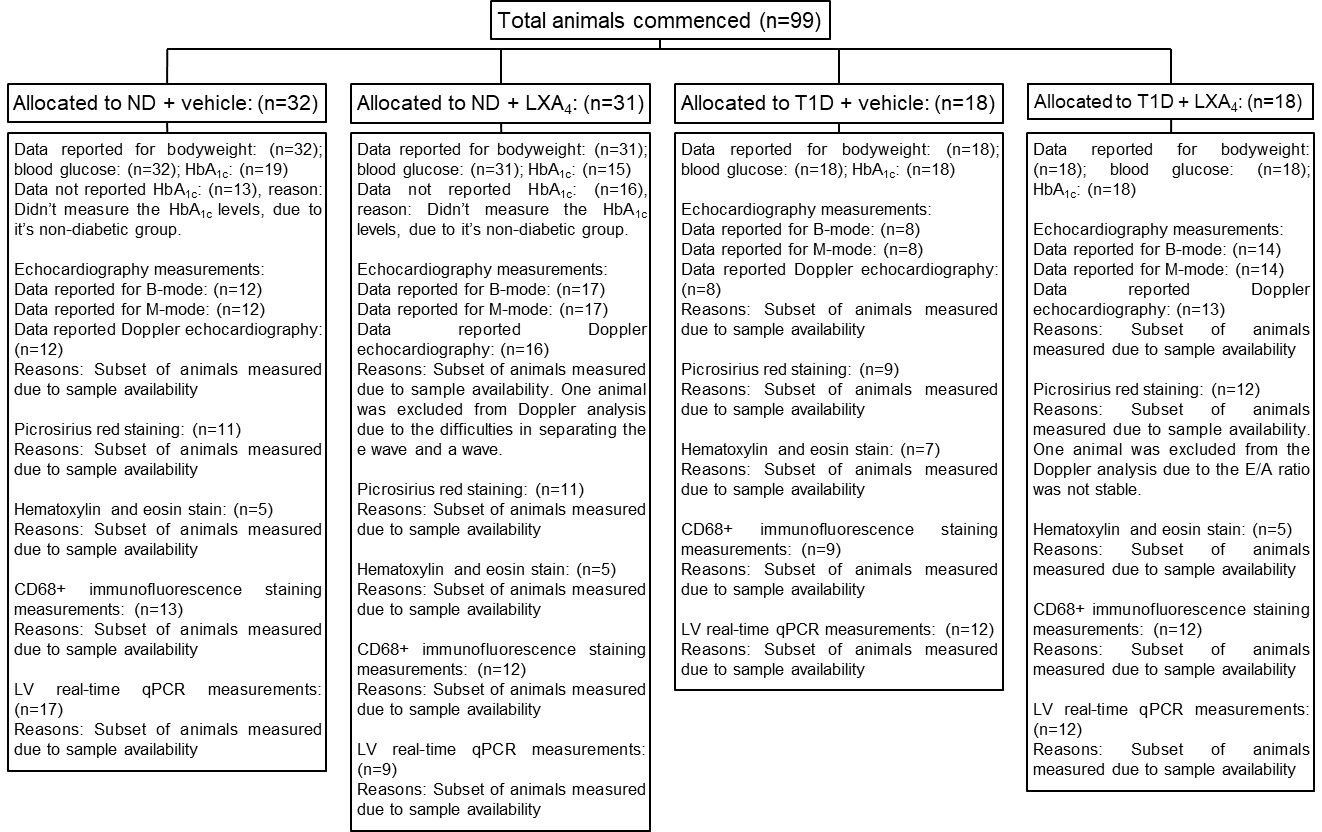
**Figure S1. Flow chart of animal.**


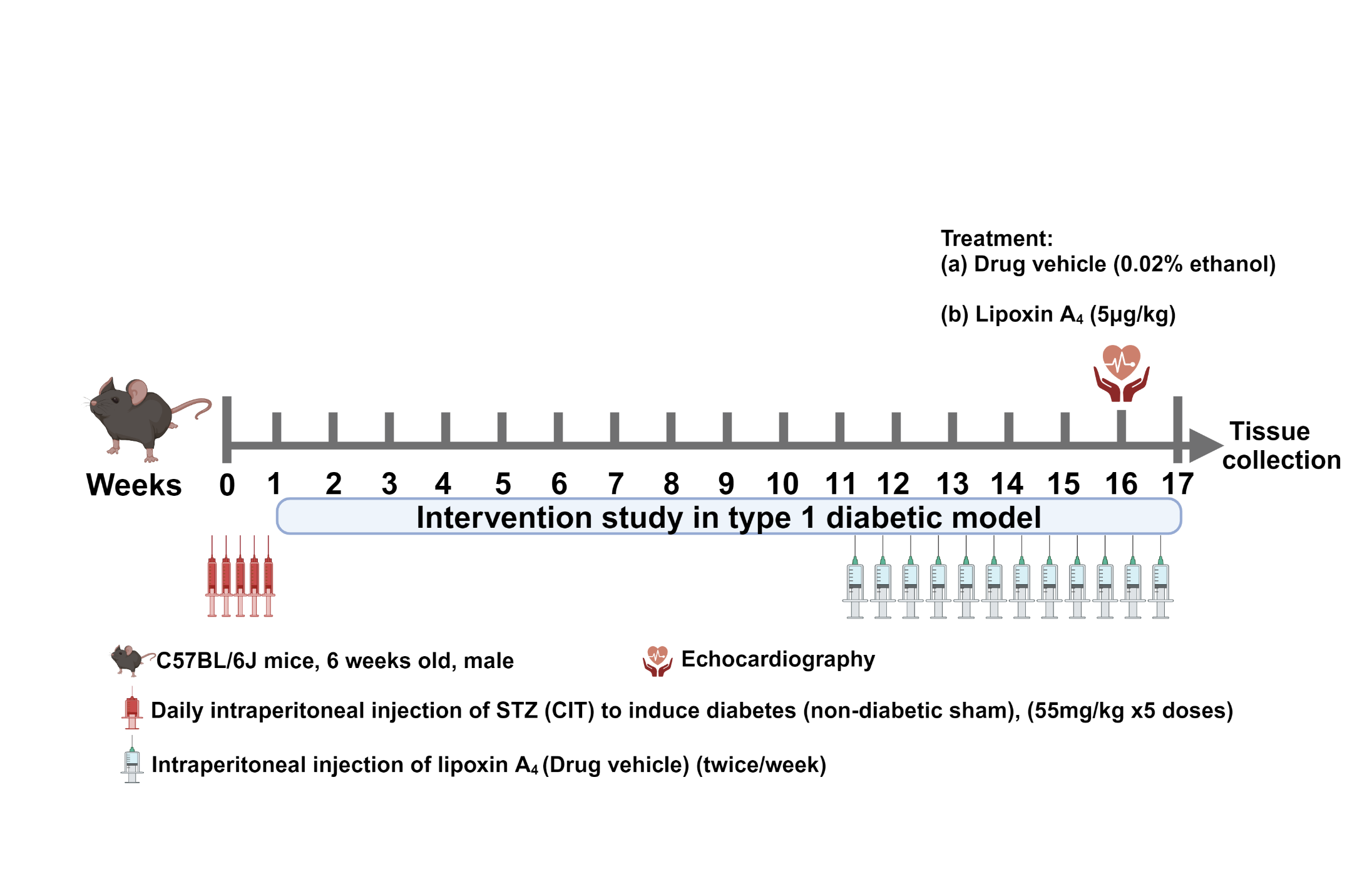
**Figure S2. Experimental design.**

**Figure S2: The experimental timeline**. Age-matched, 6-week-old ApoE^-/-^ male mice (C57BL/6 background) were randomly assigned to either the diabetic or non-diabetic cohort. The diabetic cohort received streptozotocin (STZ) for five consecutive days (i.p. 55 mg/kg/day in 0.1M citric acid vehicle). The non-diabetic cohort received citric acid vehicle by i.p. injection (200 μL of 0.5 M). Each cohort (diabetic and ND) was randomly divided into two groups, allocated to receive either drug vehicle (0.02% ethanol) or LXA_4_ (5 μg/kg, in 0.02% ethanol) twice weekly by i.p. injection. After 10 weeks of vehicle-treated diabetes, mice received LXA_4_ or vehicle from week 11 to week 16. A week before the endpoint, the echocardiography was performed. At the experimental endpoint, mouse was euthanized with an overdose of sodium pentobarbital (100 mg/kg i.p).

**2. Supplemental Tables**

**Table S1 Genes and their respective primer sequences for analysis of RNA via qRT-PCR (mouse-specific sequence)**

| Gene | Gene | Forward primer (5’-3’) | Reverse primer (3’-5’) |
| --- | --- | --- | --- |
| Actin β | *mCtβ* | CATTGCTGACAGGATGCAGAAGG | TGCTGGAAGGTGGACAGTGAGG |
| Arginase-1 | *mArg1* | TCAGAAGCTGTTCTTGGTCT | GTTCATGGGGATCCCAGTGA |
| Arachidonate 5-lipoxygenase | *mAlox5* | ATTGCCATCCAGCTCAACCA | ACTGGAACGCACCCAGATTT |
| Arachidonate 12-lipoxygenase | *mAlox12* | CAACCTAGTGCGTTTGTGGC | GGGACCACACTTGCCCC |
| Arachidonate 15-lipoxygenase | *mAlox15* | GTGTCCCCCTGATGACTTGG | CATTCCCACCACGTACCGAT |
| Connective tissue growth factor | *mCcn2* | TGACCCGGCGACCCACA | TACACCGACCCACCGAAGACACAG |
| Fibronectin | *mFn* | AAGACCATACCTGCCGAATG | CAACTGGTTGGCATGAAATG |
| Formyl peptide receptor 1 | *mFpr1* | CCTTGGCTTTCTTCA | GCCCGTTCTTTACAT |
| Formyl peptide receptor 2 | *mFpr2* | ACAGCAGTTGTGGCTTCCTT | CCTGGCCCATGAAAACATAG |
| Interleukin-1β | *mIl-1β* | TGCCACCTTTTGACAGTGATG | TGATGTGCTGCTGCGAGATT |
| Interleukin-6 | *mIl-6* | CTGGACAACATACTGCTAACCG | GGGCATCACTTCTACCAGGTAA |
| Interleukin-18 | *mIl-18* | GACTCTTGCGTCAACTTCAAGG | CAGGCTGTCTTTTGTCAACGA |
| Matrix metalloproteinase-2 | *mMmp2* | GACAAGTTCTGGAGATACAATGAAGTG | CAGGTTATCAGGCATGGCATTC |
| Matrix metalloproteinase-9 | *MMmp9* | AGACCAAGGGTACAGCCTGTTC | GGCACGCTGGAATGATCTAAG |
| Natriuretic peptide A | *mNppa* | TGGGACCCCTCCGATAGATC | AGCGAGCAGAGCCCTCAGT |
| Peroxisomal acyl-CoA oxidase type 2 | *mCox2* | CCCCCACAGTCAAAGACACT | AGTTGCTCATCACCCCACTC |
| Serum amyloid A1 | *mSaa1* | CACCAGCAGGATGAAGCTACT | CATGTCCCCAGCCCCTTG |
| S100 calcium-binding protein A9 | *mS100a9* | TCATCGACACCTTCCATCAA | GTCCAGGTCCTCCATGATGT |
| Vascular endothelial growth factor | *mVegf* | AAACGAAAGCGCAAGAAATC | ATGCTTTCTCCGCTCTGAAC |
| α-myosin heavy chain | *mMyh6* | GCTGGAAGATGAGTGCTCAGAG | CCAGCCATCTCCTCTGTTAGGT |
| β-myosin heavy chain | *mMyh7* | CCTAGACTGCAACCGAGAGG | GCAGGCTCAAGTCATCTTCC |

**Table S2 Assessment of LV systolic function via B-mode echocardiography**

|  | **Non-diabetic mice** | | **Diabetic mice** | |
| --- | --- | --- | --- | --- |
|  | **Vehicle** | **LXA_4_** | **Vehicle** | **LXA_4_** |
|  | **B-mode echocardiography** | | | |
| **n** | 12 | 17 | 8 | 14 |
| **Heart rate (bpm)** | 459 ± 13 | 468 ± 7 | 429 ± 12 | 443 ± 10 |
| **LV volume, systole (μL)** | 38.1 ± 3.0 | 39.2 ± 1.5 | 31.1 ± 2.7^#^ | 28.6 ± 1.6^$$$^ |
| **LV volume, diastole (μL)** | 71.4 ± 4.5 | 72.7 ± 2.1 | 60.0 ± 3.9^#^ | 56.8 ± 2.9^$$$^ |
| **Stroke volume (μL)** | 33.3 ± 1.8 | 33.5 ± 1.0 | 28.9 ± 1.7 | 28.1 ± 1.4^$$^ |
| **Ejection fraction (%)** | 47.7 ± 1.7 | 46.1 ± 0.8 | 48.8 ± 1.7 | 49.4 ± 0.9^$^ |
| **Longitudinal fractional shortening (%)** | 11.9 ± 0.4 | 11.5 ± 0.3 | 13.3 ± 0.5 | 13.5 ± 0.5^$$$^ |
| **Cardiac output (mL/min)** | 15.4 ± 1.0 | 15.7 ± 0.5 | 12.4 ± 0.7^#^ | 12.5 ± 0.7^$$^ |
| **LV area, systole (mm^2^)** | 18.0 ± 1.0 | 18.5 ± 0.5 | 15.8 ± 0.7 ^#^ | 15.1 ± 0.5^$$$^ |
| **LV area, diastole (mm^2^)** | 26.4 ± 1.1 | 26.8± 0.6 | 23.6 ± 0.8^#^ | 22.7 ± 0.8^$$$^ |
| Data presented as mean ± SEM. ^#^P < 0.05 vs non-diabetic + vehicle; ^$^P < 0.05, ^$$^P< 0.01, ^$$$^P< 0.001 vs non-diabetic + Lipoxin A_4_; (2-way ANOVA, Fisher’s LSD *post-hoc* for multiple comparisons). | | | | |

**Table S3 Assessment of LV systolic function via M-mode echocardiography**

|  | **Non-diabetic mice** | | **Diabetic mice** | |
| --- | --- | --- | --- | --- |
|  | **Vehicle** | **LXA_4_** | **Vehicle** | **LXA4** |
|  | **M-mode echocardiography** | | | |
| **n** | 12 | 17 | 8 | 14 |
| **Heart rate (bpm)** | 444 ± 14 | 445 ± 9 | 406 ± 11^#^ | 436 ± 10 |
| **LVPW, diastole (mm)** | 0.83 ± 0.03 | 0.84 ± 0.02 | 0.78 ± 0.04 | 0.73 ± 0.03^$$^ |
| **LVESD (mm)** | 2.72 ± 0.12 | 2.88 ± 0.06 | 2.51 ± 0.09 | 2.42 ± 0.05^$$$$^ |
| **LVEDD (mm)** | 3.96 ± 0.08 | 4.07 ± 0.05 | 3.86 ± 0.08 | 3.75 ± 0.06^$$$^ |
| **LVAW, diastole (mm)** | 0.85 ± 0.03 | 0.82 ± 0.02 | 0.76 ± 0.03 | 0.74 ± 0.02 |
| **LV mass (mg)** | 123 ± 3 | 128 ± 5 | 105 ± 7 | 94 ± 4^$$$$^ |
| **Fractional shortening (%)** | 31.6 ± 1.9 | 29.1 ± 0.8 | 35.1 ± 1.3 | 35.3 ± 1.1^$$$^ |
| Data presented as mean ± SEM. ^#^P < 0.05 vs non-diabetic + vehicle; ^$$^P< 0.01, ^$$$^P< 0.001, ^$$$$^P<0.0001 vs non-diabetic + Lipoxin A_4_ (2-way ANOVA, Fisher’s LSD *post-hoc* for multiple comparisons). | | | | |
